# Bacterial symbionts, *Buchnera*, and starvation on wing dimorphism in English grain aphid, *Sitobion avenae* (F.) (Homoptera: Aphididae)

**DOI:** 10.3389/fphys.2015.00155

**Published:** 2015-05-20

**Authors:** Fangmei Zhang, Xiangrui Li, Yunhui Zhang, Brad Coates, Xuguo “Joe” Zhou, Dengfa Cheng

**Affiliations:** ^1^State Key Laboratory for Biology of Plant Diseases and Insect Pests, Institute of Plant Protection, Chinese Academy of Agricultural SciencesBeijing, China; ^2^Corn Insects and Crop Genetics Research Unit, United States Department of Agriculture - Agricultural Research ServiceAmes, IA, USA; ^3^Department of Entomology, University of KentuckyLexington, KY, USA

**Keywords:** *Sitobion avenae*, *Buchnera*, wing dimorphism, starvation, obligate symbiont, nutrition, biotic factors, nutritional requirements

## Abstract

Wing dimorphism in aphids can be affected by multiple cues, including both biotic (nutrition, crowding, interspecific interactions, the presence of natural enemies, maternal and transgenerational effects, and alarm pheromone) and abiotic factors (temperature, humidity, and photoperiod). The majority of the phloem-feeding aphids carry *Buchnera*, an obligate symbiotic proteobacteria. *Buchnera* has a highly reduced genome size, but encode key enzymes in the tryptophan biosynthetic pathway and is crucial for nutritional balance, development and reproduction in aphids. In this study, we investigated the impact of two nutritional-based biotic factors, symbionts and starvation, on the wing dimorphism in the English grain aphid, *Sitobion avenae*, a devastating insect pest of cereal crops (e.g., wheat) worldwide. Elimination of *Buchnera* using the antibiotic rifampicin significantly reduced the formation of winged morphs, body mass, and fecundity in *S. avenae*. Furthermore, the absence of this primary endosymbiont may disrupt the nutrient acquisition in aphids and alter transgenerational phenotypic expression. Similarly, both survival rate and the formation of winged morphs were substantially reduced after neonatal (<24 h old) offspring were starved for a period of time. The combined results shed light on the impact of two nutritional-based biotic factors on the phenotypic plasticity in aphids. A better understanding of the wing dimorphism in aphids will provide the theoretical basis for the prediction and integrated management of these phloem-feeding insect pests.

## Introduction

Aphid species are soft-bodied sap-feeding insects that have developed specialized mouthparts that allow feeding on plant phloem. Although phloem contains a concentration of carbohydrates, it is nutritionally deficient in respect to nitrogenous compounds that serve as precursors to many amino acid biosynthetic pathways (Sandström and Moran, 1999). An evolutionary relationship has developed between species in insect Family Aphidoidea and the obligate intracellular endosymbiotic proteobacteria, *Buchnera aphidicola* (Lai et al., 1994). Characteristic of several other endosymbionts *Buchnera* have a highly reduced genome content that has resulted in loss of key enzymatic components of carbohydrate and lipid metabolizing pathways (Shigenobu et al., 2000), but these losses are compensated by the host-derived cellular pathways (Douglas, 1989, 1998). Correspondingly, *Buchnera* produce many essential amino acids that are not present in the nutritionally incomplete plant phloem (Douglas, 1989, 1998). Bacterial symbionts provide a supplementary source of essential nutrients, including essential amino acids (Douglas, 1989; Wilkinson and Ishikawa, 2001; Hansen and Moran, 2011), vitamins (Dadd et al., 1967; Douglas, 1988; Nakabachi and Ishikawa, 1999), and lipids (Douglas, 1988). In addition to providing nutrients, this obligate symbiosis is crucial for aphid development and reproduction (Sasaki et al., 1991; Douglas, 1995; Hardie and Leckstein, 2007; Simon et al., 2011), behavior (Hongoh and Ishikawa, 1994), pigmentation (Tsuchida et al., 2010), and resistance against biotic and abiotic stresses (Scarborough et al., 2005). This mutualism has evolved beyond nutritional interdependence, but also includes the development of the specialized adipose cells, called bacteriocytes, that are vertically transmitted from the maternal parent to offspring (Wernegreen and Moran, 2001). Phylogenetic analysis provided evidence that this symbiotic relationship could date back to at least 200 million years (Moran and Baumann, 1994; Baumann et al., 1995).

The grain aphid, *Sitobion avenae* (F.), is a destructive pest of wheat crops worldwide. As a phloem-feeding insect, aphids reduce yield by (i) sucking sap from wheat, which affects the grain filling stage, (ii) excreting honeydew which becomes covered with sooty molds, and (iii) transmitting barley yellow dwarf virus. Similar to other Aphidoidea, *S. avenae* adults can exist in either winged or wingless forms in field conditions. This phenotypic plasticity is known to arise in response to environmental cues, including changes in nutritional availability (host plant quality), overcrowding (tactile stimulation), interspecific interactions (Braendle et al., 2006 and references within), the presence of nature enemies (Weisser et al., 1999; Mehrparvar et al., 2013), and abiotic factors, such as temperature (Parish and Bale, 1990), humidity, photoperiod (Lees, 1966; Hardie, 1987), and alarm pheromone (Hatano et al., 2010). Besides these environmental cues in a traditional phenotype-by-genotype interaction, evidence suggests that maternal and trans-generational effects, including grand-maternal phenotype, maternal phenotype, and developmental stages (Müller et al., 2001) modulate wing dimorphism in aphids. This capacity of a single genotype to produce two or more discrete phenotypic forms, termed polyphenism, is somewhat prevalent across insect Orders including hymenopteran caste systems (Wilson, 1971) and morphogenesis of Lepidoptera from larval to moth forms (Shapiro, 1976).

The function of symbionts in aphid reproduction and performance has been investigated extensively (Hardie and Leckstein, 2007), however, more limited efforts have been devoted to our understanding of the impact of *Buchnera* endosymbionts on phenotypic plasticity (polyphenism) among aphid species. The objective of this research is to study the impacts of symbionts and starvation on *S. avenae* wing dimorphism. A better understanding of the mechanisms of wing development in aphids will provide new insights in the long term sustainable management of this key insect pest of wheat.

## Materials and methods

### Aphid colony maintenance

*Sitobion avenae* (F.) adults were collected from wheat fields in Langfang (39°30′42″N, 116°36′7″E), Hebei Province, China, in 2012. Aphids were maintained on 15 cm wheat seedlings in an environmental cabinet (RXZ-380B, Nb-Jn Instrument Factory, Ningbo, China) at 20°C, 60% RH, and L:D 16:8 h photoperiod. The aphid colony was generated from a single wingless adult female. The offspring from this single clone was reared in 9 cm diameter Petri dishes individually. After three generations, aphids were subjected to the subsequent experiments. The neonatal nymphs are sensitive to environmental cues associated with wing development. To generate winged morph, more than 30 newly hatched nymphs (<24 h) were reared together in 9 cm diameter Petri dishes. In contrast, only a single nymph was kept in the Petri dishes to generate wingless morph. Selective elimination of primary endosymbionts was obtained as described below.

### Rifampicin treatment

The 24 h postnatal nymphs were maintained on artificial diets (Chen et al., 2000) supplemented with 50 μg/ml of rifampicin for 48 h (Rahbe et al., 1994; Wilkinson and Douglas, 1995; Adams et al., 1996; Wilkinson and Ishikawa, 2001). This treatment has been used to eliminate aphid-*Buchnera* endosymbiotic bacteria with minimal deleterious effects on the aphid itself (Adams and Douglas, 1997). The control aphids were maintained on regular artificial diets for 48 h. The status of *Buchnera* in aphid samples was assessed by PCR. Total DNAs were isolated from natural symbiotic (control; non-rifampicin treated) and rifampicin treated individuals using E.Z.N.A.® Insect DNA Kit (OMEGA, Bio-tek) according to the manufacturer's protocol. The primers for *Buchnera*, forward primer 5′-GTCGGCTCATCACATCC-3′ and reverse primer 5′-TTCCGTCTGTATTATCTCCT-3′, were designed based on *rrl-aroE* gene sequences obtained from the *Buchnera* genome (Accession Number: GCA_000009605.1) (Shigenobu et al., 2000) using Primer 5.0 software (Primer-E Ltd., Plymouth, UK). The PCR amplification cycles included an initial denaturation step at 95°C for 5 min, 35 cycles of 95°C for 30 s, 55°C for 30 s, and 72°C for 60 s, and a final extension step of 10 min at 72°C. Amplification products were analyzed on 1% agarose gels, stained with ethidium bromide, and visualized using the Imaging G6 System (DHS Life Science & Technology Co., Ltd., Beijing, China).

### Phenotypic impacts of antibiotic treatment

Rifampicin treated and symbiotic (control) aphids were transferred to wheat seedlings, with 20 nymphs per plant. A total of five replicates were carried out for each experiment. Before the experiment, the number of winged and wingless morphs was counted, and these aphids were considered the F_1_ population (namely WG-1 and WLG-1). These adults were allowed to reproduce for 3 days. When their offspring developed into the fourth-instar nymphs or adults, the number of winged and wingless morphs were counted again, and this generation of aphids was defined as F_2_ population (namely WG-2 and WLG-2).

### Phenotypic impacts of starvation

The phenotypic impacts of starvation on wing development were assayed using first and second instar nymphs. Neonatal (<24 h) nymphs of both winged morphs were collected and kept in groups of 20 within 9 cm diameter Petri dishes provisioned with water-saturated filter papers. Nymphs were maintained on Petri dishes for 0, 24, 48, and 72 h without food before returning to wheat seedlings. The survival rate of nymphs were documented at time points 0, 24, and 48 h after treatment. When nymphs reached the adult stage, the number of winged and wingless morphs was counted, including both F_1_ and F_2_ populations.

### Statistical analysis

Statistical analyses were carried out using SAS statistical software 9.2 (SAS Institute Inc., Cary, NC, USA). Data were presented as mean ± stand error. For different treatments (antibiotics and starvation), the proportion of winged/wingless among treated and control aphids was compared and analyzed, respectively. An independent *T*-test and One-Way ANOVA for means were used to investigate the phenotypic impacts. The end point measurements included the survival rate and the percentage of winged morphs.

## Results

### Establishment of rifampicin-cured aphid lines

**Figure 1:**
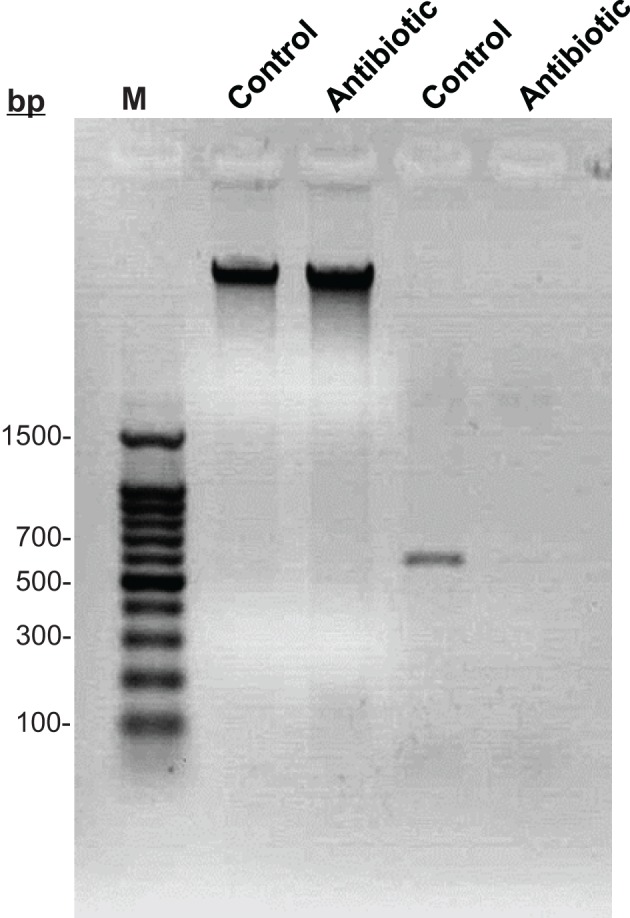
**Electrophoretic detection of *Buchnera* among symbiotic and cured aphids**. Lane 1: 100 bp ladder Marker; Lane 2: DNA of symbiotic aphids; Lane 3: DNA of rifampicin-cured aphids; Lane 4: PCR amplification of *Buchnera* in symbiotic aphids; Lane 5: PCR amplification of *Buchnera* in rifampicin-cured aphids.

To verify the elimination of *Buchnera* after antibiotic treatment, we isolated DNAs from both symbiotic and rifampicin-cured aphids, respectively. Gel electrophoresis analysis showed that the DNAs extracted from both morphs were intact (Figure 1, Lanes 2 and 3), however, *Buchnera* was absent from rifampicin-cured aphids (Figure 1, Lane 5) compared to symbiotic controls (Figure 1, Lane 4).

### Phenotypic impact of antibiotics treatment on the wing development

#### F_1_ generation

**Figure 2:**
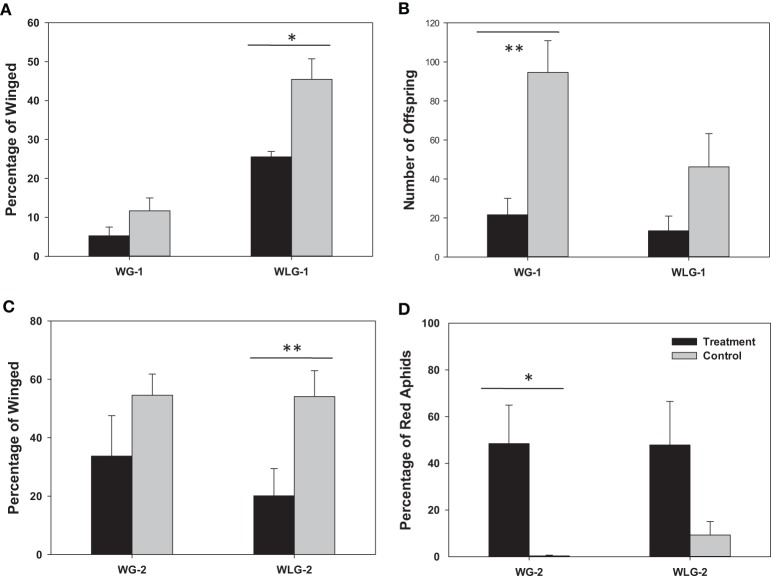
**Phenotypic impacts of rifampicin treatment on *S. avenae***. The endpoint measurements include wing development **(A)** and reproduction **(B)** in F_1_ neonatal nymphs (<24 h), and the percentage of winged morphs **(C)** and red individuals **(D)** among the neonatal F_2_ offspring (<24 h). ^*^ *P* < 0.05; ^**^ *P* < 0.01. WG-1/2: winged F_1_/F_2_ population; WLG-1/2: wingless F_1_/F_2_ population.

The overall percentage of winged morphs was reduced in rifampicin treated aphids compared to the untreated controls (Figure 2A). Specifically, 11.67 and 45.47% of the F_1_ aphids had developed wings among the *S. avenae* that were, respectively, winged and wingless before the treatment. These percentages of winged aphids were decreased to 5.25 and 25.53% in the rifampicin treatment group. For aphids derived from the parental winged group (WG-1), there was no significant difference in the wing development between the rifampicin treatment and control groups (*t* = −1.60, *P* = 0.1493), but in contrast a significant difference (*t* = −3.63, *P* = 0.0176) was observed among aphids from the initially wingless group (WLG-1). Additionally, significant differences in the proportion of winged and wingless individuals, were detected between WG-1 and WLG-1 for both, the rifampicin (*t* = −7.62, *P* = 0.0001) and the control treatments (*t* = −5.40, *P* = 0.0006). Elimination of *Buchnera* from neonatal nymphs significantly reduced subsequent fecundity in aphids compared to control groups (Figure 2B). The number of offspring is 94.60 and 46.20 among winged and wingless *S. avenae* before the treatments. These numbers dropped to 21.60 and 13.40 after rifampicin treatment. For the initially wingless individuals (WLG-1), no significant differences were detected between the number of offspring produced in the rifampicin-treated and the control groups (*t* = −1.74, *P* = 0.1194), but significant differences were detected between treatments for the initially winged individuals (WG-1) (*t* = −3.98, *P* = 0.0041).

#### F_2_ generation

**Figure 3:**
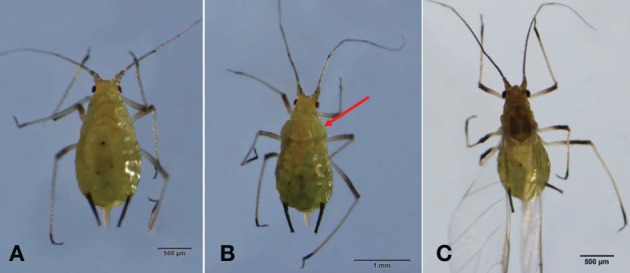
**Morphological changes of adult *S. avenae* after rifampicin treatment. (A)** a wingless adult; **(B)** an intermediate form which should have developed into a winged morph but, instead, retained the sclerotized thorax and wing remnants (arrow); **(C)** a winged adult.

The percentage of winged morphs was also reduced in the F_2_ generation among rifampicin-treated aphids compared to the untreated controls (Figure 2C). Specifically, 54.54 and 54.11% of the F_2_ aphids had wings, respectively, among winged (WG-2) and wingless *S. avenae* groups in the untreated controls (WLG-2). These percentages dropped to 33.72 and 20.14% after rifampicin treatment for WG-2 and WLG-2, respectively. For winged individuals (WG-1), there were no significant difference in the proportion of individuals that developed wings between the rifampicin-treated and the untreated controls (*t* = −1.33, *P* = 0.2200). In contrast, significant differences were observed for the wingless group (WLG-2) (*t* = −3.81, *P* = 0.0052). The proportion of a red colored morph was not significantly different between WG-2 (48.44%) compared to WLG-2 (47.85%) (*t* = 0.02, *P* = 0.9815), although a significant difference was observed in the percentage of the red color between rifampicin-treated and untreated controls in WG-2 (*t* = 2.92, *P* = 0.0433). No significant differences in the red colored morph were detected among treatments in the WLG-2 (*t* = 1.98, *P* = 0.1079) (Figure 2D). It is worth noting that, besides the normal wingless (Figure 3A) and winged (Figure 3C) forms, a new apterous adult form in the rifampicin-treated aphids showed an intermediate winged/wingless phenotype (Figure 3B).

#### Between generations

The percentage of winged morphs between generations exhibited a different trend. Specifically, the percentage of winged morphs was 5.25 and 33.7% for WG-1 and WG-2, respectively, while it was 25.53 and 12.53% in the rifampicin treated groups. Moreover, there were no significant differences in the development of wing dimorphism between the generations (WG-1 vs. WG-2: *t* = −2.03, *P* = 0.1089; WLG-1 vs. WLG-2: *t* = 1.99, *P* = 0.1109).

### Phenotypic impact of starvation on the wing development

**Figure 4:**
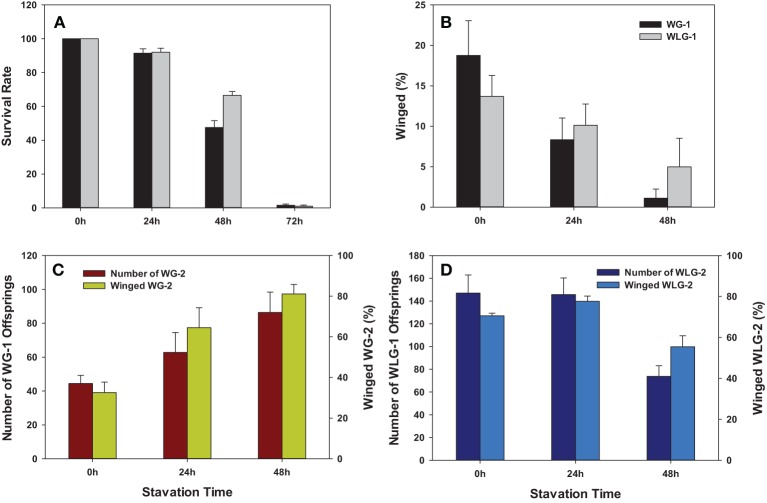
**Phenotypic impacts of starvation treatment on *S. avenae***. The endpoint measurements include **(A)** Survivorship; **(B)** wing development; and production of F_1_ and winged individuals among F_2_ offspring from winged **(C)** and wingless **(D)** adults. WG-1/2: winged F_1_/F_2_ population; WLG-1/2: wingless F_1_/F_2_ population.

Both survival rate and the percentage of winged morphs were significantly reduced after starvation treatment to neonatal nymphs (Figures 4A,B). Starvation for 0, 24, 48, and 72 h had significantly different mean survival rates in WG-1 (*F* = 341.83, *P* < 0.0001) and WLG-1 (*F* = 630.16, *P* < 0.0001), respectively. The survival rates were consistently over 90% when aphids were starved for 24 h, with no significant difference between WG-1 and WLG-1. In contrast, survivorships in WG-1 and WLG-1 were reduced substantially to 47.5 and 66.5%, respectively, after aphids were starved for 48 h. The survival rate decreased dramatically to 1.5 and 1.0% in, respectively, WG-1 and WLG-1 after 72 h starvation, at which point the proportion of individuals that had developed wings could no longer be effectively estimated. Aphid mortality reached 100% when starved for more than 72 h. Consequently, wing development was further investigated only for aphids starved 0, 24, and 48 h.

Starvation significantly reduced the percentage of the winged morphs in F_1_ *S. avenae*. Specifically, the percentage of winged aphids was drastically reduced from 18.75 to 8.33 and 1.11%, respectively, after 24 and 48 h of starvation treatments in WG-1 (*F* = 8.75, *P* = 0.0045). However, the percentage of winged morphs in WLG-1 was reduced from 13.69 to 10.12 and 4.97%, respectively, after 24 and 48 h of starvation treatment, with no significant differences between different starvation times (*F* = 2.20, *P* = 0.1530) (Table 1, Figure 4B). Among the three different starvation times, there were no significant differences in the wing development between WG-1 and WLG-1 (0 h: *t* = 1.01, *P* = 0.3434; 24 h: *t* = −0.48, *P* = 0.6473; 48 h: *t* = −1.00, *P* = 0.3738) (Table 1).

**Table 1:** **The number of offspring and proportion of the winged individuals derived from the winged and wingless adults**.

	**The proportion of F_1_**		**The number of offspring of F_1_**		**The proportion of F_2_**	
	**WG-1**	**WLG-1**	**WG-2**	**WLG-2**	**WG-2**	**WLG-2**
0 h	18.75 ± 4.3^a^	13.69 ± 2.58^a^	44.4 ± 4.87^a^	147 ± 15.97^a^	32.50 ± 5.2^a^	70.54 ± 1.32^a^
24 h	8.33 ± 2.69^b^	10.12 ± 2.64^a^	62.8 ± 11.74^ab^	145.8 ± 14.43^a^	64.50 ± 9.84^b^	77.66 ± 2.58^a^
48 h	1.11 ± 1.11^b^	4.97 ± 3.55^a^	86.4 ± 12.09^b^	73.8 ± 9.4^b^	81.12 ± 4.64^b^	55.44 ± 5.36^b^

*Values are mean ± SE. Means within a column followed by different letters (a, b) are significantly different (LSD's test: P < 0.05)*.

The starvation to neonatal nymphs affected aphid fecundity in comparison to the non-starved controls. Specifically, the number of offspring produced for the WG-2 generated increased from 44.4 to 86.4 when WG-1 parents starved 0 and 48 h were compared. The opposite effect was shown in a decrease of WLG-2 offspring from 147.0 to 73.8 when contrasting those derived from WLG-1 parents starved 0 and 48 h (Figure 4C). This change in reproduction (number of offspring) across time (0 vs. 48 h) was reflected as a significant difference in WG-2 (*F* = 4.32, *P* = 0.0386) and WLG-2 (*F* = 9.70, *P* = 0.0031) (Table 1). On the contrary, the proportion of the winged aphids increased as the starvation time was prolonged in WG-2 (*F* = 12.60, *P* = 0.0011) (Figure 4D). There were no significant differences in the percentage of winged individuals between controls and 24 h starvation treatments in WLG-2. Starvation for 48 h led to significantly lower rate of winged offspring compared to the non-starved controls and 24 h-starved aphids (*F* = 10.04, *P* = 0.0257) (Table 1).

## Discussion

Symbiotic relationships with intracellular bacteria, such as *Wolbachia* and *Buchnera*, are pronounced across Insecta, and have evolved intricate interdependencies for mutual survival and propagation (Baumann et al., 2000; Werren et al., 2008). Many insect endosymbionts have crucial roles in host survival, whereby removal of these symbionts often results in notable fitness costs. Previous studies on *Buchnera*, the primary symbionts of sap-feeding insects, has revealed several effects on host insect fitness, development and reproduction (Sasaki et al., 1990; Douglas, 1995, 1998; Febvay et al., 1995). Furthermore, detailed information available indicated the impact of aposymbionts on the performance, nutrition, metabolism, and feeding behavior of aphid (Prosser and Douglas, 1991; Wilkinson and Douglas, 1995, 1996). Moran and Yun (2015) found that aphids with *Buchnera* replacement exhibited a dramatic increase in their heat tolerance. This relationship with *Buchnera* is essential for compensation of nutritional deficiencies in plant phloem encountered by sap-feeding insects, and forms the basis of this mutualism. Thus aphids, through the mutualistic association with *Buchnera* symbionts have attained the capacity to utilize phloem sap as the sole dietary source throughout their lifecycle (Sasaki et al., 1990; Douglas and Prosser, 1992; Febvay et al., 1995). It was shown that 10 essential amino acids are scarce in the phloem sap (Sandström and Pettersson, 1994). Analysis and annotation of the *Buchnera* genome predicted the presence of most genes involved in the biosynthesis of essential amino acids and may lack certain cell wall lipid biosynthetic pathways (Shigenobu et al., 2000; Tamas et al., 2002; van Ham et al., 2003), and thus are supplied to the host through the endosymbiontic relationship (Baumann, 2005). Indeed, genome analysis reveals host–symbiont cooperation in the production of amino acids in aphids (Hansen and Moran, 2011). These prior analyses strongly suggested that that *Buchnera* are capable of synthesizing essential amino acids required that for aphid nutritional health, but may be depending on aphids in order to obtain structural lipids. Furthermore, the development and maternal transmission of specialized adipose cells, bacteriocytes, to offspring (Wernegreen and Moran, 2001) show that host cell and reproductive modifications have occurred during this intricate co-evolution. It can be argued that symbiosis resulted in the co-evolution aphids and *Buchnera* genomes in order to facilitate this partnership and this interaction could have allowed for the emergence of the aphid branch of hemipteran insects greater than 200 million years ago (Moran and Baumann, 1994; Baumann et al., 1995).

Prior studies have demonstrated that nutrient availability affects wing dimorphism in aphids and is one of the factors that influence this polyphenism, whereby nutritional deficiencies increase the proportion of apterous (winged) progeny (Johnson, 1966; Müller et al., 2001; Braendle et al., 2006). *Buchnera*, being an obligate intracellular symbiont with a relatively well-understood role in providing essential amino acids to aphid hosts, may play a role in wing dimorphism. Specifically, it was revealed that the seemingly obvious consequences of removing the *Buchnera* endosymbiont from aphids by antibiotic treatment had decreased the propensity of aphids to develop as winged individuals. Specifically, prior studies showed that feeding bean seedling treated with chlortetracycline caused a majority of *Aphis fabae* to develop as wingless or winged/wingless intermediate adult forms (Leonardo and Mondor, 2006). The primary *Buchnera* symbiont of *Acyrthosiphon pisum* has very similar effects on aphid fitness and polyphenism (Russell and Moran, 2006; Hardie and Leckstein, 2007). Data from the current study clearly demonstrated *Buchnera*-free neonatal *S. avenae* resulted in a significant reduction in the proportions of winged offspring, and, in agreement with prior studies indicated above, suggested that the primary endosymbiont can affect wing dimorphism in Aphidoidea. Although intriguing to surmise a direct effect between *Buchnera* and wing dimorphism, a highly likely explanation for these and other results may be that the resulting lack of essential amino acids and reduced aphid vigor could be the cause of this reduction in winged forms (Hardie and Leckstein, 2007). Indeed, previous studies investigated the effect of amino acids on the wing dimorphism in aphids, and showed that the deletion of methionine, isoleucine, or histidine, respectively, could significantly increase the percentage of winged morphs in *Myzus persicae* (Dadd, 1968; Dadd and Krieger, 1968; Mittler and Kleinjan, 1970; Sutherland and Mittler, 1971). Similarly, the development of winged individuals in *A. fabae* was induced by the removal of dietary arginine, leucine, lysine, and proline, respectively (Leckstein and Liewellyn, 1973).

Our results showed a highly significant interaction between *Buchnera* and the development of winged individuals in the treatments WG-1 and WLG-1. Previous studies evidencing that the features of nutritional interactions between the aphid and its symbionts were substantially varied by aphid's species, age and morph (Douglas and Dixon, 1987). The possible explanations for this phenomenon were suggested to involve the switching off/on of the genes involved in the formation of wing tissues or wingless development (Hardie and Leckstein, 2007). Under the nutritionally adverse conditions part of mycetocytes are decreased (Hongoh and Ishikawa, 1994), causing the changes of number and size of the symbiont and further wing development changes. However, the effect of aposymbiotic on wing dimorphism in aphids was not applied to any species, such as *A. pisum* and *M. viciae* (Hardie and Leckstein, 2007). Elimination of bacteria with antibiotic rifampicin from *S. avenae* significantly reduced growth sterility, and reduced aphid mass in almost all cases, especially reproduction of F_1_ of winged adults, which is consistent with previous reports (Douglas, 1995, 1998; Wilkinson and Douglas, 1995; Douglas, 1995; Hardie and Leckstein, 2007).

Moran and Jarvik (2010) observed that aphid genome contained several genes for carotenoid synthesis, which were not found in animal genomes, and might be responsible for red or green coloration of the aphids. Aphid body color can be affected by many factors such as temperature, host plant, light, symbionts, and natural enemies (Braendle et al., 2006 and references within). Intriguingly, our data showed that the number of aphids with red coloration increased significantly when the aphid were subjected to bacteria-free treatment, and cleared of the *Buchnera* endosymbiont. It represents the first known study to show this direct interaction. Previous studies showed that the coinfecting of facultative endosymbiont, *Rickettsiella* and *Hamiltonella*, could change the body color of pea aphid (*A. pisum*) from red to green (Tsuchida et al., 2010, 2014). These changes in aphid body color can influence prey-predator interactions by changing visual cues and camouflage. Although upregulation of carotenoids may be a cause of the change in aphid coloration, further studies are required to document molecular genetic changes in this pathway at the transcriptional level and/or determine the involvement of *Buchnera* (if any) in mediating these phenotypic change.

Starvation is a common experience of natural populations which causes individuals to respond to this stressful conditions by utilizing various adaptive strategies (Karan and David, 2000). In this study, starvation was used to mimic the nutritional deficiency caused under natural conditions, and resulted in a relative decrease in the formation of winged individuals as a function of increased duration of starvation among F_1_ individuals for both WG-1 and WLG-1 laboratory populations. Interestingly, both the rate of reproduction in the F_1_ (as measured by the number of WG-1 and WLG-1 progeny) and the proportion of winged individuals in the F_2_ generation (WG-2 and WLG-2) were significantly increased with prolonged periods of starvation. Refeeding after the starvation also led to an increase in numbers and size of mycetocytes in aphids. Prior studies showed that starved aphids exhibited a drastic decrease in the total volume of mycetocyte, which was reversed by refeeding. This observed decrease could result from host utilization of nutrients within the mycetocytes as the expense of *Buchnera* provisioning (Hongoh and Ishikawa, 1994), or could also be a consequence of reduced *Buchnera* titers as host nutrients become increasingly scares during periods of starvation. Of greater importance, our results showed that a transgenerational effect of starvation, whereby the proportion of winged individuals produced in WG-2 and WLG-2 were directly related to the duration of starvation experienced by the maternal WG-1 and WLG-1. Many insects employ multiple reproductive strategies that enable short-term tolerance of a lack of food resource, a lack of suitable oviposition sites or other environmental stresses (Brough and Dixon, 1990; Xu et al., 2012). Some reports indicated that prolonged periods of starvation for both parent aphids and young can favored the development of wingless individuals (Johnson, 1966; Xu et al., 2012). Thus, our laboratory experiments demonstrate that unfavorable nutritional conditions (starvation) influence wing dimorphism by increasing the proportion of mobile winged individuals in the subsequent generation, and may represent an adaptive mechanism which promotes dispersal and resulting utilization of new food resources.

Combined results from both starvation and aposymbiosis studies indicate a significant role of symbionts in the wing dimorphism in aphid, which may be related to their production of essential amino acids. Additional investigations are warranted to understand the molecular signaling and interactions between symbionts and wing dimorphism in aphids. A better understanding of the wing development in aphids will provide novel targets for the sustainable management of this phloem-feeding insect pest.

## Author contributions

Conceived and designed the experiments: XL. Performed the experiments: XL and FZ. Analyzed the data: XL, FZ, and XZ. Contributed reagents/materials/analysis tools: XL, YZ, and DC. Wrote the paper: XL, FZ, BC, and XZ. All authors read and approved the final manuscript.

### Conflict of interest statement

The authors declare that the research was conducted in the absence of any commercial or financial relationships that could be construed as a potential conflict of interest.
